# The Consequences of Biofilm Dispersal on the Host

**DOI:** 10.1038/s41598-018-29121-2

**Published:** 2018-07-16

**Authors:** Derek Fleming, Kendra Rumbaugh

**Affiliations:** 1 Department of Surgery, Texas Tech University Health Sciences Center, Lubbock, Texas 79430 USA; 2 Department of Immunology and Molecular Microbiology, Texas Tech University Health Sciences Center, Lubbock, Texas 79430 USA; 3 Department of the TTUHSC Surgery Burn Center of Research Excellence, Texas Tech University Health Sciences Center, Lubbock, Texas 79430 USA

## Abstract

Chronic infections are often associated with the presence of a biofilm, a community of microorganisms coexisting within a protective matrix of extracellular polymeric substance. Living within a biofilm can make resident microbes significantly more tolerant to antibiotics in comparison to planktonic, free-floating cells. Thus, agents that can degrade biofilms are being pursued for clinical applications. While biofilm degrading and dispersing agents may represent attractive adjunctive therapies for biofilm-associated chronic infections, very little is known about how the host responds to the sudden dispersal of biofilm cells. In this study, we found that large-scale, *in vivo* dispersal of motile biofilm bacteria by glycoside hydrolases caused lethal septicemia in the absence of antibiotic therapy in a mouse wound model. However, when administered prudently, biofilm degrading enzymes had the potential to potentiate the efficacy of antibiotics and help resolve biofilm-associated wound infections.

## Introduction

Chronic infections are often exacerbated by the presence of a biofilm, a complex community of microorganisms living within a matrix of polysaccharides, proteins, eDNA, lipids and other molecules that comprise the extracellular polymeric substance (EPS). Living within the protection of the EPS, one or multiple species of microbes are afforded greatly increased tolerances to both antimicrobials and host defenses^1,2^. Biofilm-associated tolerance is due to several proposed mechanisms. The EPS provides a physical barrier that can be difficult for antibiotics to penetrate, and bacteria within the biofilm often display reduced metabolic activity, which greatly influences their susceptibility to antibiotics, the majority of which depend on active metabolism^3^. Thus, biofilm infections, which have been estimated to include 80% of all human bacterial infections, and 90% of chronic wound infections^4,5^, are highly recalcitrant to traditional therapies.

As an alternative approach to directly targeting the causative pathogens of a biofilm infection, many researchers have directed their efforts towards degrading EPS matrix constituents^6^. In theory, dispersal of biofilm microbes into their planktonic form will increase their susceptibility to antimicrobials and the host immune system. Further, because they do not directly target the microorganisms themselves, they should be less likely to drive resistance. To date, a host of EPS-specific dispersal agents have been investigated, with targets including, but not limited to, structural exopolysaccharides, exoproteins, and eDNA^3,6^. However, it should be noted that clinical application of such therapies are virtually non-existent, with the exception of Dornase alpha (Pulmozyme) as an FDA-approved therapy for the breakup of DNA-rich mucus presenting in cystic fibrosis patients^7,8^, but which also may be active on biofilms in the lungs. While medically induced dispersal of a mature biofilms *in vivo* has yet to be demonstrated, EPS-targeting, especially enzymatic-mediated deconstruction of matrix constituents, represents a promising antibiofilm avenue.

Exopolysaccharides are one of the major structural components for the majority of EPS producers^9^. They play a variety of vital roles in biofilm formation and persistence, including but not limited to: surface and cell-cell adhesion and aggregation, tolerance to desiccation, mechanical stability, nutrient sorption and storage, binding of enzymes, and physical protection against antimicrobials and the environment^9^. Considering their ubiquity and importance to the structural integrity of the EPS matrix, active degradation of exopolysaccharides represents a promising approach to clinically eradicating biofilm infections. To target biofilm exopolysaccharides, we have employed glycoside hydrolases (GHs), which are enzymes that hydrolyze the glycosidic linkages between two or more carbohydrates^10^. They can be individually characterized by the specific type of linkage that they cleave, such as α-1,4 bond hydrolysis by α-amylase, β-1,4 bond hydrolysis by cellulase, or β-1,3 bond hydrolysis by β-1,3 galactosidase^11,12^. By targeting common, highly conserved glycosidic linkages, a single therapy can potentially prove efficacious against the EPS produced by a broad-spectrum of pathogens, and against the highly complex and compositionally chimeric polymicrobial biofilms often seen clinically^13^. Additionally, GHs are unlikely to pose significant risk to patients, because their targets (glycosidic linkages) are not readily found in human tissue.

In our previous work, we showed that a dual-enzyme combination of α-amylase and cellulase resulted in significant reductions in biomass, and the dispersal of biofilm-dwelling bacteria, allowing for an increase in the effectiveness of antibiotic treatments *in vitro*^14^. However, it has been hypothesized that triggering a large-scale dispersal event in a living host can overwhelm the immune system, causing dissemination of the infection and possibly lethal septicemia^15^. To our knowledge, biofilm dispersal-induced septicemia has never been demonstrated, and in this study we show that: (1) Large-scale dispersal can result in a lethal septic event (2) the development of septicemia appears to be dependent on the motility of the dispersed bacteria (3) the probability of death by septicemia is positively correlated to wound size (4) concurrent systemic and topical antibiotics can protect the host against septicemia (5) GH therapy augments the ability of antibiotic intervention to clear the infection.

## Results

### Glycoside hydrolases disperse biofilms *in vivo*, but cause rapid septicemia

**Figure 1:**
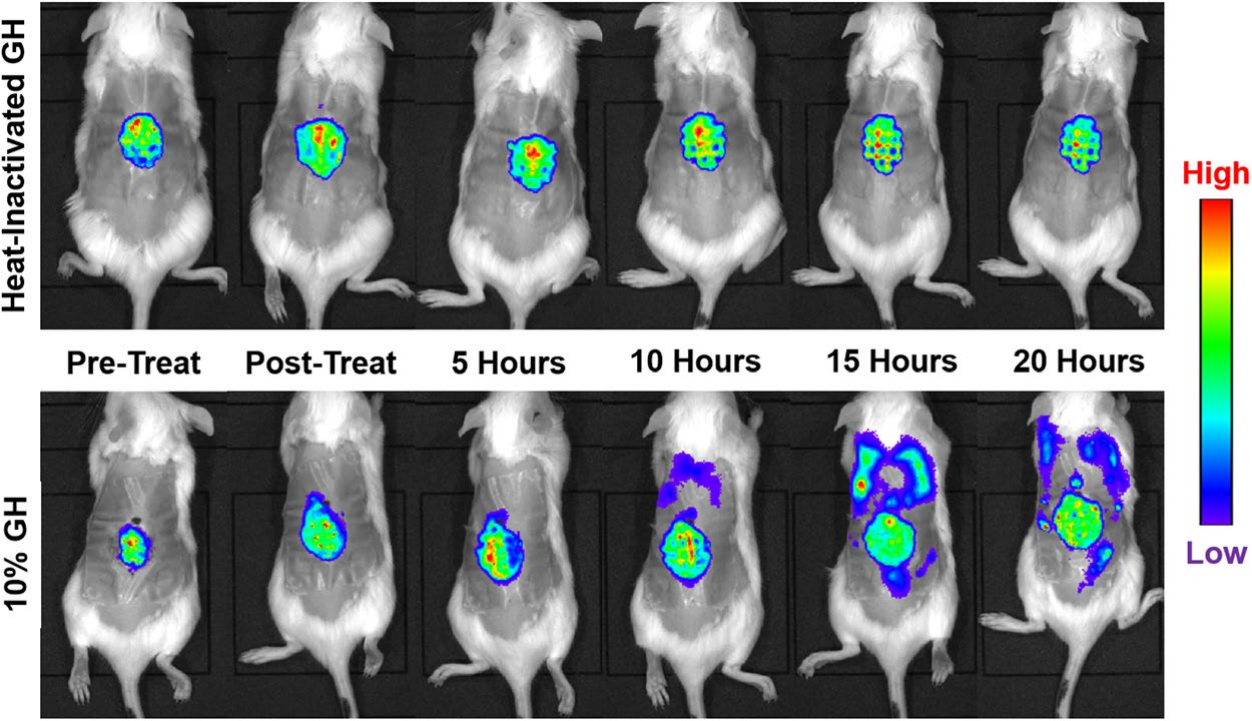
IVIS imaging of *in vivo* dispersal triggered by glycoside hydrolase therapy. Treatment of 48-hour-old mouse chronic wounds, infected with bioluminescent *P. aeruginosa*, with 10% α-amylase and cellulase (1:1; GH), or heat-inactivated control, resulted in dispersal and systemic spread of the infection. Clear localization of bacteria in the organs can be seen in the treated group. A representative animal from the treatment and control groups at each time point are shown.

It has been hypothesized that inducing a substantial dispersal event *in vivo* may overwhelm the host immune system, possibly resulting in fatal septicemia^3^. When we treated 2-day-old *S. aureus* and *P. aeruginosa* wound infections *in situ* with 10% GH, it triggered the dispersal of more than 10^8^ cells (Supplemental Fig. 1), resulting in significant septicemia within as short as 15 hours (Fig. 1**)**. Pre-treating with 10% GH prior to inoculation did not cause an increase in septicemia, indicating that the effect of GH that influences bacteremia is directly on the biofilm and indirectly not on the host tissue or vasculature (Supplemental Table 1). The systemic spread of bacteria appeared to occur via the cardiovascular system, with detectable levels of bacteria appearing in the blood in as little as 5 hours post-treatment (as determined by whole-blood colony forming unit quantification on selective agar; Supplemental Fig. 2). To our knowledge, we are the first to show that fatal septicemia can be induced by large-scaled dispersal of a biofilm infection.

### Dispersal-mediated septicemia is dependent upon swimming-motility

**Figure 2:**
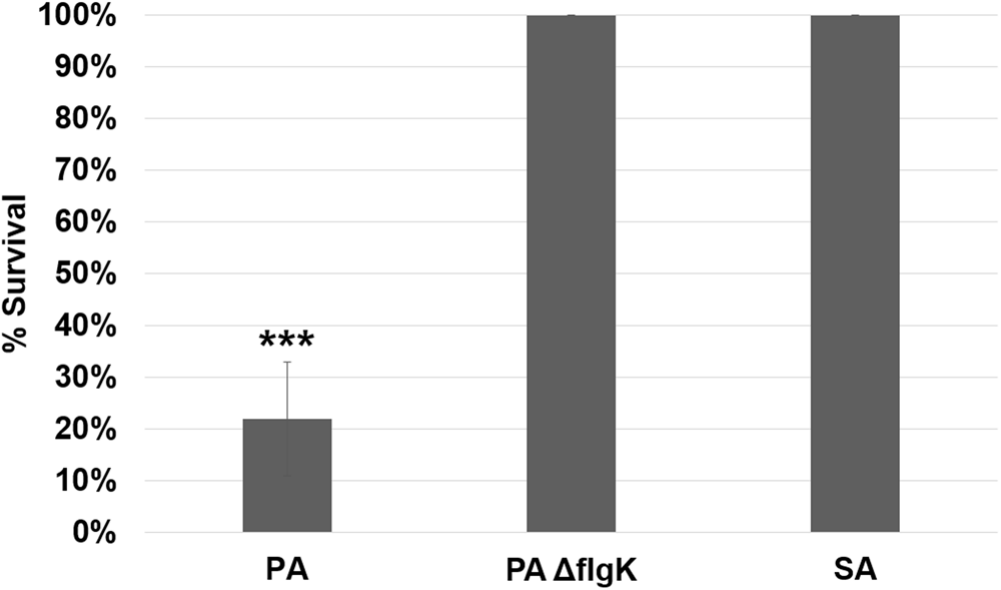
Swimming motility is required for dispersal-induced septicemia. Treatment of 48-hour mouse chronic wounds, infected with wild-type (PA) or a flagella mutant of *P. aeruginosa*, or with *S. aureus* (SA), with 10% α-amylase and cellulase (1:1; GH), resulted in ~80% septicemia only in mice infected with the motile strain. Repeated measures ANOVA and the Tukey-Kramer multiple-comparison test were used to test for differences between columns: ***p < 0.001. N = 9 for each group.

In order to investigate and characterize the pathogenesis of fatal septicemia brought on by biofilm dispersal, we first investigated whether bacterial motility was correlated with increased rates of septicemia. Mouse wound beds were inoculated with a motile *P. aeruginosa* wild-type strain, a non-motile *P. aeruginosa* flagellar mutant, or the non-motile Gram-positive wound pathogen, *S. aureus* (SA31). Infections were allowed to establish over 48 hours, and then wounds were treated with GHs, and mice were monitored over 36 hours for septicemia. Only infection with the motile *P. aeruginosa* wild-type strain proved fatal, with nearly 80% of the animals becoming moribund (Fig. 2) after GH treatment. This difference was not due to bacterial load, as there was no significant difference between the bacterial load in the wounds of mice infected with the different strains (average pre-treatment CFU/g of wound tissue: PA = 4.33 × 10^7^, PA ΔflgK = 3.00 × 10^8^, SA = 1.90 × 10^7^; significance determined via one-way ANOVA and the Tukey-Kramer multiple-comparison test). Thus, it would appear that swimming motility is necessary for dispersal-induced septicemia in this wound infection model. That is, given that we have already shown that *S. aureus* is dispersed from the biofilm at a similar level to *P. aeruginosa* (see Supplementary Fig. 1), it is likely that non-motile bacteria are unable to escape the confines of the wound-bed.

### Dispersal-mediated septicemia is positively correlated with wound size

**Figure 3:**
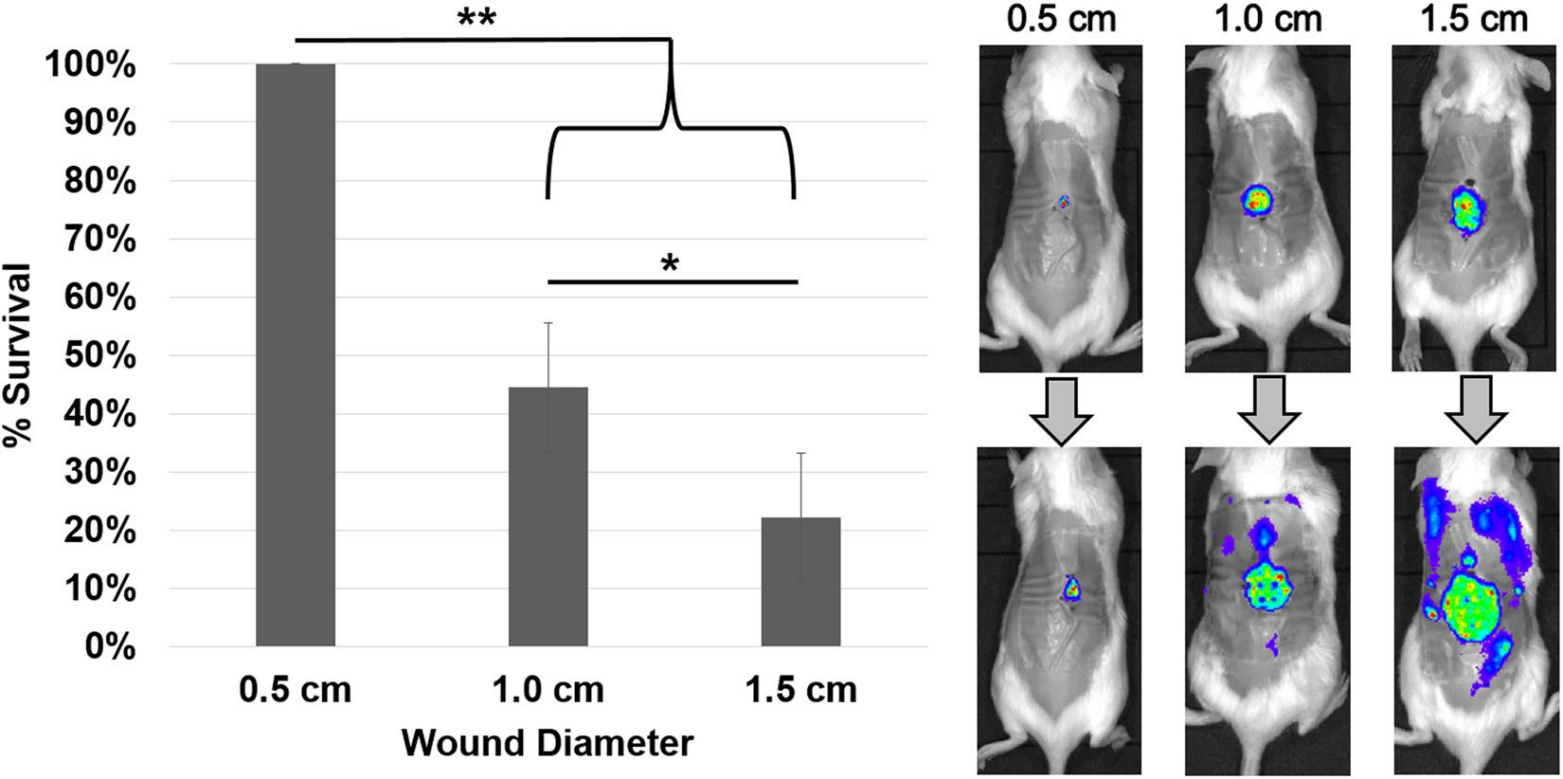
Wound size is positively correlated with dispersal-induced septicemia. Treatment of 48-hour mouse chronic wounds of varying sizes (0.5, 1.0, and 1.5 cm in diameter) with 10% α-amylase and cellulase (1:1; GH) resulted in no mortality in the 0.5 cm group after 36 hours, and significantly increased mortality in the 1.0 cm and 1.5 cm groups, indicating that increased wound size is correlated with septicemia. A representative mouse for each wound size, before and after treatment, is shown on the right, with arrows indicating treatment. Repeated measures ANOVA and the Tukey-Kramer multiple-comparison test were used to test for differences between columns: *p < 0.05, **p < 0.01. N = 9 for each group.

To determine the effect of wound surface area on dispersal-induced septicemia, mice were administered wounds of varying diameters, including 0.5 cm, 1.0 cm, and 1.5 cm. The wounds were infected with bioluminescent *P. aeruginosa*, after which biofilms were allowed to grow over 48 hours. The established infections were treated with 10% GH as above, and the animals were monitored over 36 hours for septicemia. No septicemia occurred for the smallest wound size (0.5 cm diameter), while mice with the medium wound size (1.0 cm diameter) exhibited a mortality rate of greater than 50%, and mice with the largest wound size (1.5 cm diameter) nearly 80% (Fig. 3). No significant differences in bacterial load were observed between the small and medium wounds, or between the medium and large wounds (average CFU/gram wound tissue: small = 1.03 × 10^7^, medium = 5.33 × 10^7^, large = 2.00 × 10^8^; significance determined via one-way ANOVA and the Tukey-Kramer multiple-comparison test), indicating that greater surface area, and not increased bacterial load, was more correlated with bacteremia.

### Antibiotics protect against dispersal-mediated septicemia, and are potentiated by concurrent glycoside hydrolase therapy

**Figure 4:**
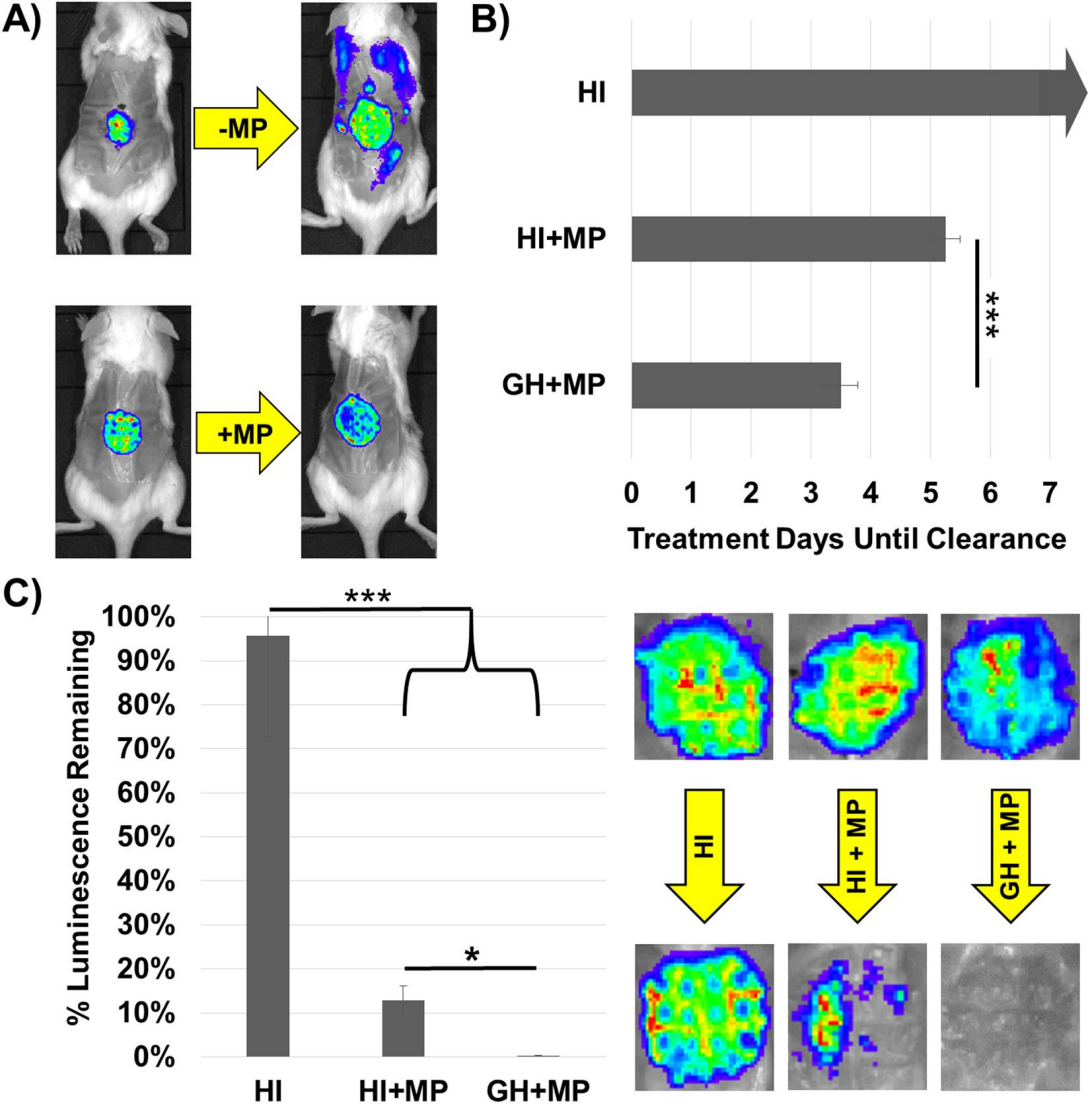
Antibiotics protect against dispersal-induced septicemia, and GH treatment augments antibiotic efficacy. Topical or systemic (pictured) meropenem (MP) protected against 10% α-amylase and cellulase (1:1; GH) dispersal-induced septicemia (**A**). Wounds were treated with heat-inactivated (HI) enzyme control, with or without concurrent topical MP (3 mg/ml) every 24 hours for 3 days, starting at hour zero. GH therapy significantly improved infection clearance vs. MP and HI enzyme. Treatment days until clearance” refers to the number of days until luminescence was reduced to zero (**B**). Luminescence quantification and representative IVIS images of wound-beds on treatment day 3 showed complete clearance of enzyme plus antibiotic treated wounds (**C**). One-way ANOVA and the Tukey-Kramer multiple-comparison test were used to test for differences between columns: *p < 0.05 ***p < 0.001. N = 9 for each group.

To test if concurrent systemic or topical antibiotics can protect against dispersal-mediated septicemia, 48-hour wound infections comprised of bioluminescent *P. aeruginosa* were treated with 10% GH as above, and monitored via an *in vivo* imaging system (IVIS) for systemic spread. Both topical (3 mg/ml) and systemic (300 mg/kg) administration of meropenem prevented dissemination of *P. aeruginosa* (Fig. 4A). In addition to protecting the host from bacteremia, daily measurement of luminescence signal loss prior to daily treatments with heat-inactivated 10% GH alone, heat-inactivated GH plus topical meropenem (3 mg/ml), and 10% GH plus meropenem revealed that infection clearance occurred significantly faster for the GH plus meropenem group (Fig. 4B,C). Meropenem was chosen as a clinically-relevant, broad-spectrum antibiotic that doesn’t contain either linkage targeted by the α-amylase and cellulase mixture. It should also be noted that meropenem, like most antibiotics, targets only metabolically active cells, suggesting that the newly-dispersed cells are in fact metabolically active.

## Discussion

With a plethora of dispersal agents currently in development and in pre-clinical testing, the question has been raised as to what effect triggering a large-scale release of biofilm microbes will have on the patient^3,6^. Christensen *et al*. showed that reducing cyclic-di-GMP levels in implant-associated *P. aeruginosa* biofilms via induction of phosphodiesterase activity caused a temporary but seemingly harmless accumulation of bacteria in the spleen^16^. However, potentially fatal systemic spread resulting from substantial biofilm dispersal has never been reported.

We previously showed that α-amylase and cellulase, glycosidases that hydrolyze highly conserved linkages in EPS polysaccharides, can significantly degrade polymicrobial biofilms containing *S. aureus* and *P. aeruginosa*, resulting in the dispersal and antibiotic sensitization of the bacteria^14^. Here we show that GH-induced biofilm dispersal can trigger a substantial release of bacteria into the host (with bacteria detectable by blood culture within as little as 5 hours), and can lead to fatal bacteremia. Further investigation revealed that the dispersal-induced septicemia was dependent on bacterial motility and overall wound size. It should be noted, however, that 1.0 cm- and 1.5 cm diameter wounds are roughly equivalent to 12.5% and 22% total body surface area (TBSA) respectively (based on Meeh’s formula; TBSA in m^2^ = 9.83 × [weight in kg × 1 o000]^2/3^/10000), or 18% based on the Wallace rule of nines^17^, and a similar sized chronic wound is unlikely for the majority of human cases. For example, the entire foot is less than 2% TBSA (rule of nines), thus a typical diabetic foot ulcer would represent less than 1%.

Another factor to consider regarding the risk of dispersal-induced septicemia is that patients with a chronic biofilm infection will most likely be on an antibiotic regimen^18,19^, and as GH treatment of biofilms is a non-bactericidal approach^14^, it would most likely be implemented as an adjunctive therapy to normal antibiotic administration. We observed that concurrent treatment with both systemic and topical meropenem protected mice from dispersal-mediated septicemia. Furthermore, GH treatment potentiated topical meropenem activity and significantly decreased the time required to clear the infection. Thus, simultaneous GH plus antibiotic therapy represents a prospective new approach to managing chronic biofilm infections.

It should also be noted that the GH administration utilized in this study was a rapid, high-dose treatment regimen designed to trigger a significant dispersal event over a short period of time. That being said, a more controlled administration of the enzymes to the biofilm, in the form of extended release hydrogels or loaded wound bandages, for example, could represent another safeguard against systemic spread. By dispersing the biofilm microbes at low levels over an extended period of time, the immune system would be afforded a better opportunity to eradicate the newly liberated cells. Other *in vivo* studies have shown that slow dissolution of the biofilm matrix is able to avoid the complication of systemic dissemination. For example, monoclonal antibodies against DNA binding proteins have been found to trigger the extended degradation of established *P. aeruginosa* biofilms in various murine models of biofilm infection with no signs of dispersal-induced death^20,21^.

In conclusion, the ability of a large-scale dispersal event, induced by therapeutic intervention with a dispersal agent, to lead to a potentially fatal septicemic event is of significant concern. Given the breadth of research currently underway into the development of clinically applicable dispersal agents^3,6^, these findings could potentially impact many areas of medical biofilm research, from chronic wounds, to sinusitis, indwelling devices and more. Any agent which has the potential to disperse a large quantity of motile cells should be investigated for safety in relevant animal models, and the need for concurrent antibiotic administration, or other safeguards, such as the controlled release of the dispersal agent to the biofilm, should be determined.

## Methods

### Bacterial strains

*P. aeruginosa* wild-type strains PAO1^22^ and MPAO1^23^, *S. aureus* wild-type strain SA31^24^, a MPAO1 *flgK* transposon mutant (PW2960; PA1086-F09::IS*lacZ*/hah)^23^, and a bioluminescent PAO1 strain carrying the luminescence reporter plasmid pQF50-lux^25^ have all been previously described. All *S. aureus* and *P. aeruginosa* strains were grown in baffled Erlenmeyer flasks, with shaking at 200 rpm, in Luria-Bertani (LB) broth at 37 °C. Planktonically grown cells were then used to initiate infection in the *in vivo* model. All colony forming units (CFU) were quantified by serial dilution and 10 μL spot-plating on Staphylococcus Medium 110 (Difco) and Pseudomonas Isolation Agar (Difco).

### Mouse model

Our murine surgical excision wound model has been previously described^14,26–29^. Briefly, mice were anesthetized by intraperitoneal injection of sodium pentobarbital. After a surgical plane of anesthesia was reached, the backs were shaved and administered a full-thickness, dorsal excisional skin wound to the level of panniculus muscle with surgical scissors. Wounds were then covered with a semipermeable polyurethane dressing (Opsite dressing; Smith & Nephew), under which 10^4^ bacterial cells were injected into the wound-bed. Biofilm formation was allowed to proceed for 48–72 hours, a time at which we have demonstrated the presence of biofilm in wounds^24,28,30^.

### Glycoside hydrolases and antibiotics

Bacterial alpha-amylase (from *Bacillus subtilis*; MP Biomedicals) and fungal cellulase (from *Aspergillis niger*; MP Biomedicals) were utilized for these experiments. Briefly, powdered enzymes were dissolved in phosphate-buffered saline to achieve the desired percentage concentration (w/v). Heat inactivation was performed by heating the enzyme solutions for 25 minutes at 90 °C.

Established infections were treated via topical application of vehicle control, heat-inactivated GH, antibiotic alone (or in combination with heat-inactivated GH), GH alone, or GH plus antibiotic (meropenem). Briefly, wound beds were irrigated with a 10% α-amylase and cellulase (in a 1:1 combination) solution in three separate topical infusions with 30 minutes of dwell time for each, with or without antibiotics. Septicemia was determined by monitoring animals for signs of systemic illness (lethargy, loss of appetite, labored breathing, tremors), and moribund mice were euthanized via phenobarbital injection. *In vivo* cell dispersal was determined by collecting the dispersed cells in the post-treatment irrigation solution, plating on selective agar, and quantifying cells dispersed by treatment vs. control solutions. In order to image dispersal *in vivo*, we utilized a Lumina II XR *In Vivo* Imaging System (IVIS). Mice with established wound infections were administered GH therapy, and imaging occurred immediately following treatment, and every 4-5 hours subsequently. For experiments involving systemic antibiotic therapy, peritoneal dosing of 300 mg/kg meropenem occurred 4 hours prior to, and 8 hours following GH treatment. For topical antibiotic therapy, 5 mg/mL meropenem was added to the GH or control treatment solutions.

### Vertebrate animal use

All animal experiments were carried out in strict accordance with the recommendations in the Guide for the Care and Use of Laboratory Animals of the National Institutes of Health. The protocol was approved by the Institutional Animal Care and Use Committee of Texas Tech University Health Sciences Center (Protocol Number: 07044).

### Data availability

All data generated or analyzed during this study are available upon request to the corresponding author.

## Electronic supplementary material

Supplementary Data
