# Supplementary material for: The Consequences of Biofilm Dispersal on the Host
Source: Sci Rep. 2018 Jul 16;8:10738. doi: 10.1038/s41598-018-29121-2 (PMC6048044; doi:10.1038/s41598-018-29121-2)
Supplement: Supplementary file 1 — Supplementary Data [file 41598_2018_29121_MOESM1_ESM.pdf]

The Consequences of Biofilm Dispersal on the Host

Derek Fleming[1,2] and Kendra Rumbaugh[1,2,3,*]

[1]Departments of Surgery[1], Immunology and Molecular Microbiology[2], and the TTUHSC Surgery Burn Center of Research Excellence[3], Texas Tech University Health Sciences Center, Lubbock, Texas, 79430, U.S.A.
*Kendra.Rumbaugh@ttuhsc.edu

Supplementary Data

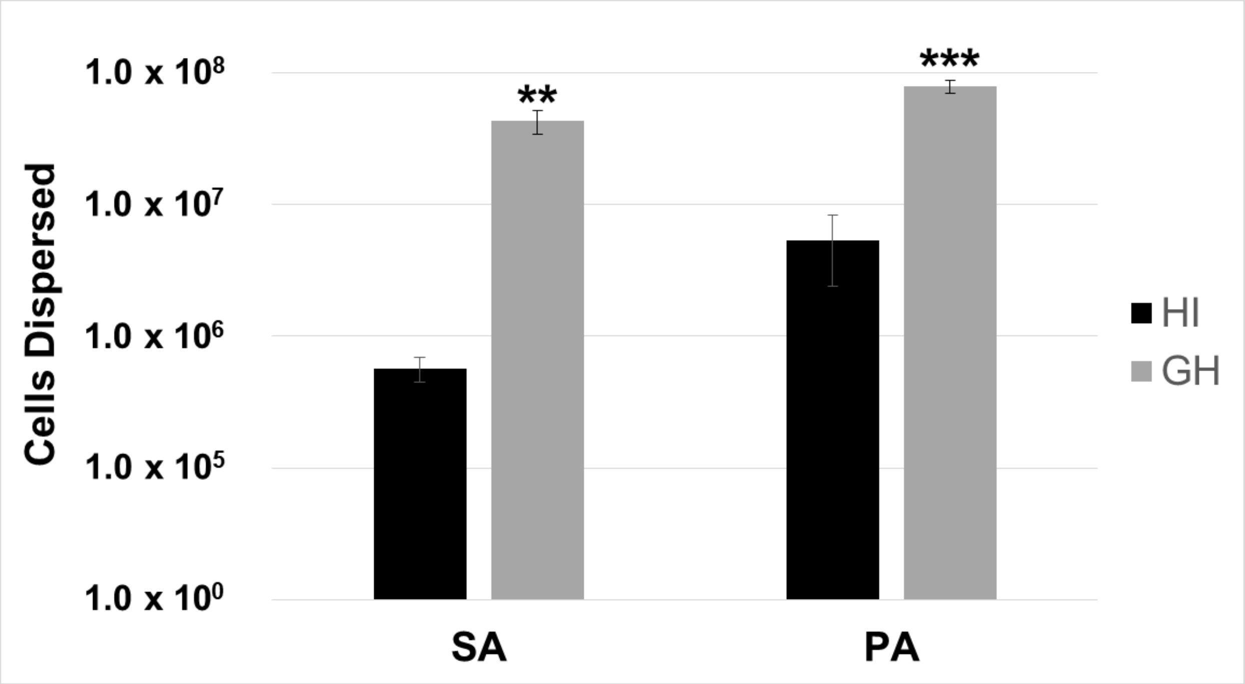

Supplementary Figure 1. *In vivo* GH-induced dispersal quantification. Treatment of 48-hour mouse chronic wounds, infected with *S. aureus* and *P. aeruginosa*, with 10% α-amylase and cellulase (1:1; GH) resulted in significant dispersal of bacterial cells from the biofilms into the post-treatment irrigation solution in comparison to heat-inactivated enzyme controls (HI). One-way ANOVA and the Tukey-Kramer multiple-comparison test were used to test for differences between columns: **$p < 0.01$ ***$p<.001$. N=3 for each group.

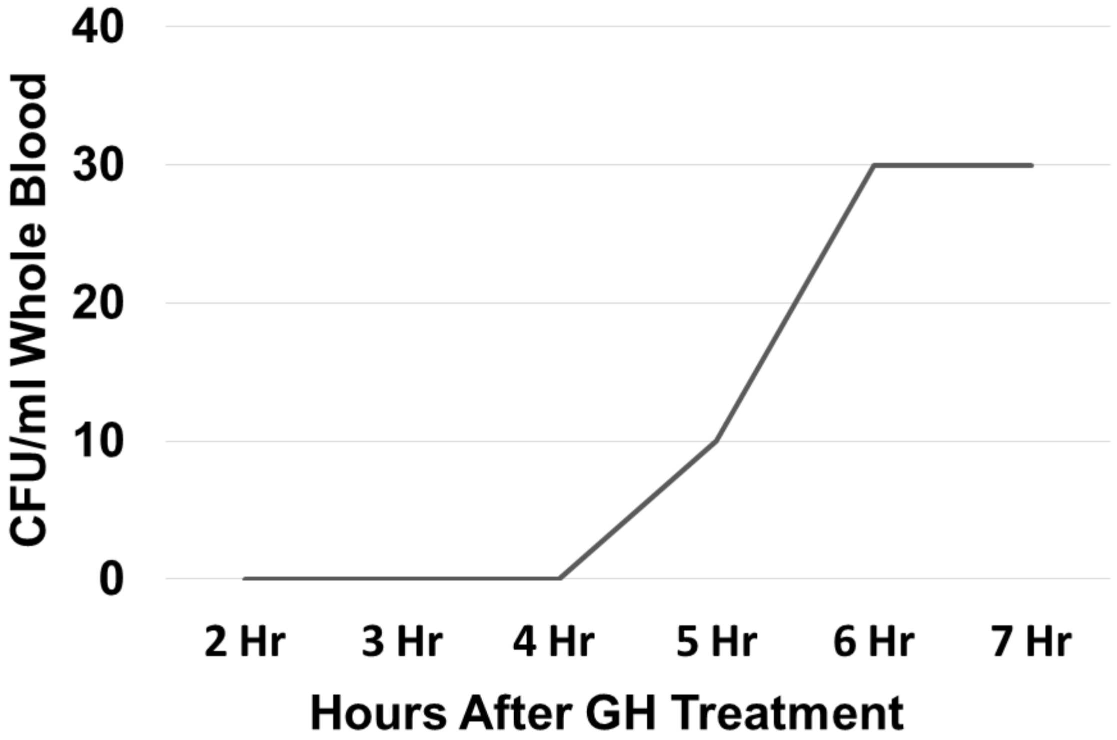

Supplementary Figure 2. Systemic spread of dispersed bacteria occurs through the bloodstream. Treatment of a representative 48-hour mouse chronic wound, infected with *P. aeruginosa*, with 10% α-amylase and cellulase (1:1; GH) resulted in a detectable load of bacteria within extracted whole, venous blood as detected by quantification on selective agar (Pseudomonas Isolation Agar).

	Spleen CFU	Liver CFU	Kidney CFU
**PBS 1**	0	0	0
**PBS 2**	0	0	0
**PBS 3**	2.0×10^3	2.0×10^3	1.0×10^3
**GH 1**	0	0	0
**GH 2**	0	0	0
**GH 3**	0	0	0

Supplementary Table 1. Pre-infection treatment with GH does not induce septicemia. Treatment of mice with 10% α-amylase and cellulase (1:1; GH), compared to vehicle control (PBS), prior to infection with *P. aeruginosa* did not render the animals susceptible to septicemia. N=3 for each treatment type. Organs were harvested 48 hours after treatment for quantification of colony forming units (CFU).
